# Effect of neurokinin B and dynorphin A on kisspeptin-10 secretion from the anterior pituitary cells of pubescent ewes *in vitro*

**DOI:** 10.2478/jvetres-2025-0026

**Published:** 2025-05-03

**Authors:** Natalia Szysiak, Urszula Kosior-Korzecka, Monika Greguła-Kania, Krzysztof Patkowski, Mateusz Fila, Andrzej Junkuszew

**Affiliations:** 1 Sub-Department of Pathophysiology, Department of Preclinical Veterinary Sciences, Faculty of Veterinary Medicine, University of Life Sciences in Lublin, 20-033 Lublin, Poland; 2 Department of Animal Breeding and Agricultural Advisory, Faculty of Animal Sciences and Bioeconomy, University of Life Sciences in Lublin, 20-950, Lublin, Poland

**Keywords:** neurokinin B, dynorphin A, kisspeptin, pituitary, puberty

## Abstract

**Introduction:**

Neurokinin B (NKB), dynorphin A (Dyn A) and kisspeptin (KiSS) are key agents that participate in the neuroendocrine regulation of the development and functioning of the reproductive system. While the role of KiSS is better understood, the functions of NKB and Dyn A at the pituitary level have not been elucidated. The objective of our study was to analyse their direct effect on kisspeptin-10 (KiSS-10) secretion by anterior pituitary cells isolated from pubescent ewes.

**Material and Methods:**

Pituitary cells from 10-month-old ewe lambs were incubated in McCoy’s 5A medium without hormones (the control), or with 10^−11^, 10^−10^, 10^−9^, 10^−8^ or 10^−7^ M of NKB or Dyn A for 2, 4, 6, 12, 18 or 24 h. The concentration of KiSS-10 was analysed by ELISA using species-specific antibodies.

**Results:**

When applied at the concentrations of 10^−10^–10^−7^ M, NKB increased KiSS-10 secretion throughout the entire experiment (2–24 h), compared to the control. Significantly higher (P-value ≤ 0.05) KiSS-10 release than in the control was observed after 6–24 h exposure of the cells to 10^−8^ M of NKB. However, no effect of NKB on the secretion of KiSS-10 was shown when applied at the lowest concentration (10^−11^ M). In turn, there was no significant effect of Dyn A at any concentration on KiSS-10 secretion by pituitary cells at any time

**Conclusion:**

In contrast to Dyn A, NKB can directly affect KiSS-10 secretion from the pituitary cells of pubescent ewes in a way dependent on the time of exposure to this neuropeptide and its concentration in the culture medium. This phenomenon may indicate a potential role of NKB in the initiation of reproductive activity, which leads to the achievement of sexual maturity in the optimal developmental window.

## Introduction

It is known that delayed puberty in livestock may have negative health effects, including an increase in the amount of subcutaneous and visceral fatty tissue and obesity, and may consequently have the negative economic effect of reduced productivity (1). Reaching sexual maturity depends on many factors, *e.g*. sufficient production of neuronal agents like kisspeptin-10 (KiSS-10), neurokinin B (NKB) and dynorphin A (Dyn A) by KNDy (kisspeptin, neurokinin B and dynorphin) neurons in the central nervous system. These three neuropeptides play a relevant role in the neuroendocrine regulation of the development and functioning of the reproductive system where they regulate ovulation and fertility (17). As a 10-amino-acid peptide belonging to the tachykinin family, NKB directly stimulates hypothalamic KNDy neurons, leading to secretion of kisspeptins (13). These are a family of neuropeptides and endogenous ligands of G protein-coupled receptors (GPR54). Kisspeptins binding to their receptor on gonadotropin-releasing hormone (GnRH) neurons in the preoptic area (POA) and arcuate nucleus (ARC) in the hypothalamus enhance the pulsatile secretion of GnRH, which results in luteinising hormone (LH) and follicle-stimulating hormone (FSH) secretion in the anterior pituitary gland, mediating the NKB effect (17). In turn, dynorphins, belonging to the family of endogenous opioid peptides produced in the ARC, inhibit prepubertal GnRH secretion and consequently gonadotropin secretion by the anterior pituitary gland (5). The KiSS-1/GPR54 and NKB/neurokinin B receptor (NK3R) systems are key factors in the hormonal regulation of puberty onset. It is known that mutations in the *TAC3* or *TACR3* genes, respectively coding for NKB or NK3R, cause hypogonadotropic hypogonadism in humans because of disturbances in the secretion of gonadotropic hormones from the anterior pituitary gland (21, 27, 30). As shown by Li *et al*. (9), KiSS-10 and senktide (an NK3R agonist) infused into the lateral ventricle during the luteal phase of the ovine oestrous cycle increased the LH pulse frequency and the mean LH levels (9). They also reported that in conditions of minimal GnRH/LH secretion, central NK3R agonist infusion induced LH release, similarly to the response to KiSS-10. Moreover, Goodman *et al*. (6) demonstrated that the ablation of over 90% of KNDy neurons in the ARC of the hypothalamus caused by application of MePhe7-NKB (a selective NK3 receptor agonist) conjugated to saporin resulted in a decrease in the LH pulse amplitude in ovariectomised adult Blackface ewes (6). In addition, the expression of GPR54 in the pituitary gland was found, which proves that a KiSS can act directly at this level, affecting the secretion of tropic hormones (19, 22, 23, 31). Single data indicate that NKB and Dyn A may also be involved in the synthesis and secretion of hormones in the pituitary gland (10, 11, 12, 28). However, to the best of our knowledge, there are no reports on the direct effect of NKB or Dyn A on KiSS secretion from the ovine pituitary gland. Therefore, the aim of this study was to analyse, for the first time, the influence of NKB and Dyn A on KiSS secretion by pituitary cells isolated from pubescent ewes.

## Material and Methods

### Experiment design

The protocol of the experimental design and all procedures were approved by the Local Ethics Committee for Animal Experimentation in Lublin (No. 65/2023). The cell culture was prepared using pituitary glands isolated from 10-month-old ewe lambs of the Polish Lowland sheep, Uhruska variety (n = 6; mean body weight: 39.52 ± 3.25 kg), housed at the Professor T. Efner Small Ruminant Research Station in Bezek (Poland) in the autumn season. The ewes were humanely euthanised by electric shock and exsanguinated at a local slaughter house in accordance with applicable regulations. The pituitary glands were dissected and transported within 1 h to the laboratory at approximately 3–5°C in Dulbecco’s Modified Eagle’s Medium (DMEM) supplemented with 0.59% 4-(2-hydroxyethyl)-1-piperazineethanesulfonic acid (HEPES), 0.08% glucose, 0.1% bovine serum albumin and gentamicin at 20 μg/mL. The anterior and posterior lobes of the pituitary were separated by blunt dissection. The anterior pituitary tissue was minced and repeatedly enzymatically digested with 0.25% trypsin for 10 min each time at 37°C. After each digestion round, the cells were washed three times in DMEM and centrifuged at 1,200 rpm for 10 min. After the last centrifugation, the pituitary cells were passed through a 60-μm nylon filter and counted in a Bürker chamber. Cell viability evaluated by 0.4% trypan blue dye exclusion was higher than 96%. The pituitary cells at 250,000 cells/mL were then resuspended in McCoy’s 5A medium containing 2.5% foetal calf serum, 10% horse serum, 0.59% HEPES, a mixture of amino acids and vitamins, and gentamicin at 20 μg/mL, and the solution was adjusted to pH 7.4 and seeded in 24-well culture plates in 1 mL aliquots per well. The cells were allowed to attach for 96 h at 37°C under a 5% CO_2_ atmosphere (8, 18, 19, 24) until the start of the experiments. After attachment to the dishes and formation of a monolayer, the pituitary cells were incubated in McCoy’s 5A medium without hormones (the control), or with 10^−11^, 10^−10^, 10^−9^, 10^−8^ or 10^−7^ M of NKB or Dyn A. After 2, 4, 6, 12, 18 or 24 h of the experiment, the media were collected and stored at -20°C to determine the cumulative concentration of KiSS-10 by ELISA using species-specific antibodies (KiSS-1 (112–121) Amide/Kisspeptin-10/Metastin 45–54) Amide EIA (enzyme immunoassay) Kit; Phoenix Pharmaceuticals, Burlingame, CA, USA). The kisspeptin secretion was expressed as the concentration (ng/mL) of the hormone released into the culture medium by 250,000 cells during the particular incubation time.

### Statistical analysis

The results were calculated using Statistica 13.0 PL (Dell, Round Rock, TX, USA) and expressed as a mean and standard deviation (x ± SD). Comparisons between the control and experimental cultures were performed using analysis of variance and paired *t*-tests. Differences with a P-value ≤ 0.05 were considered significant. Pearson linear correlation coefficients were calculated to assess the relationships between the analysed variables: NKB concentration or Dyn A concentration and KiSS-10 secretion.

## Results

### Influence of NKB on KiSS-10 secretion by ovine pituitary cells *in vitro*

**Fig. 1.**
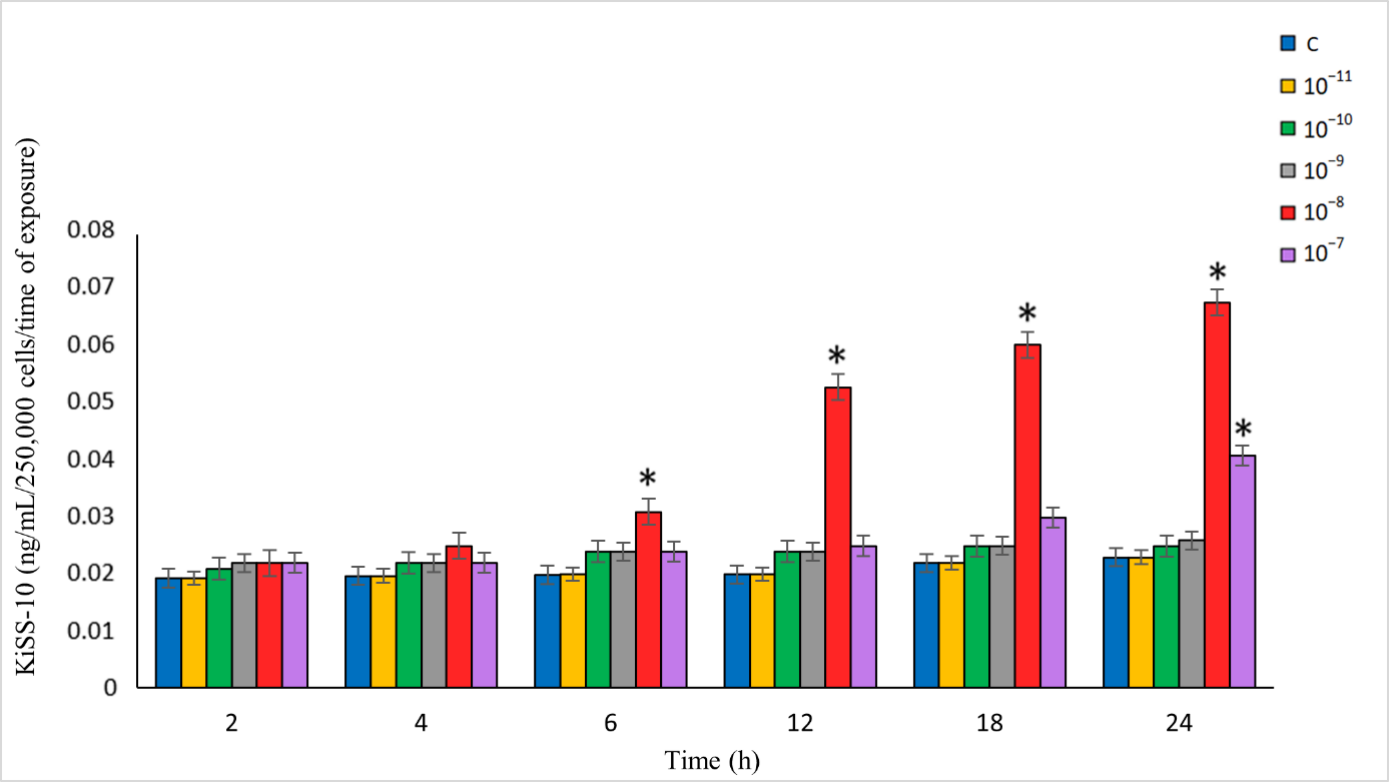
Influence of neurokinin B on kisspeptin-10 (KiSS-10) secretion from ovine pituitary cells *in vitro* * – statistically significant difference (P-value ≤ 0.05) compared to the control

The effect of NKB on KiSS-10 secretion was dependent on the time of exposure and the NKB concentration in the culture medium (which ranged over 10^−11^–10^−7^ M). The treatment of the cells with 10^−10^–10^−7^ M of NKB resulted in elevated KiSS-10 secretion throughout the entire experiment compared to the control. A significant effect of NKB (P-value ≤ 0.05) on KiSS-10 was found after 6–24 h at the dose of 10^−8^ M and after 24 h at 10^−7^ M of NKB. The KiSS-10 release reached a maximum after the 24-h exposure of the cells to NKB at the concentration of 10^−8^ M. This value was significantly higher (P-value ≤ 0.05) than in the control and in the other cultures with NKB. However, no effect of NKB at the concentration of 10^−11^ M was observed on the secretion of KiSS-10 (Fig. 1).

There was no correlation between the concentration of NKB and KiSS-10 secretion from the ovine pituitary cells after 4, 6, 12, 18 or 24 h (r = 0.04, r = 0.01, r = –0.08, r = 0.01 and r = 0.23, respectively). However, a low positive correlation was found between the NKB concentration over the full range in the experiment and the KiSS-10 secretion after 2 h (r = 0.4). In turn, there was a moderate positive correlation between NKB in the range of 10^−11^–10^−8^ M and KiSS-10 secretion after 2 h (r = 0.55), a high positive correlation after 4 h (r = 0.89) and a very high positive correlation after 6, 12, 18 and 24 h (r = 0.93, r = 0.99, r = 1.0 and r = 1.0, respectively).

### Influence of Dyn A on KiSS-10 secretion by ovine pituitary cells *in vitro*

**Fig. 2.**
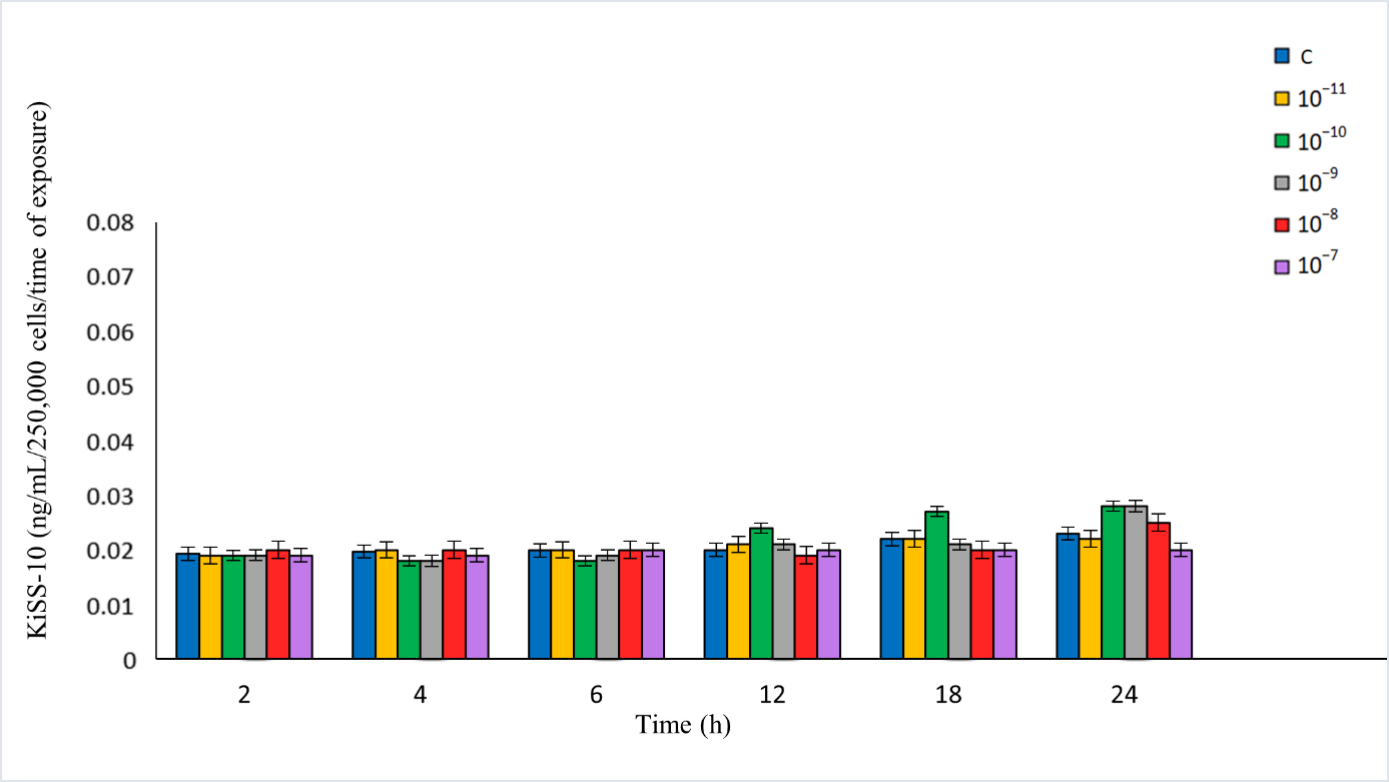
Influence of dynorphin A on kisspeptin-10 (KiSS-10) secretion from ovine pituitary cells *in vitro*

There was no significant effect of Dyn A applied in the full concentration range over the whole time of the experiment on the secretion of KiSS-10 by the ovine pituitary cells. However, Dyn A used at 10^−10^ M caused a slight increase in KiSS-10 after 12 and 24 h, and used at 10^−9^–10^−8^ M, it did so after 24 h, compared to the control and the other cultures with Dyn A (Fig. 2). There was no correlation between the concentration of Dyn A in the range of 10^−11^–10^−7^ M and KiSS-10 secretion from the ovine pituitary cells after 2 and 4 h (r = –0.16 and r = 0.05, respectively). However, the study showed a low positive correlation between the concentration of Dyn A over the full range in the experiment and KiSS-10 secretion after 6 h (r = 0.42), a low negative correlation after 12 and 18 h (r = –0.37 and r = –0.43, respectively), and a high negative correlation after 24 h (r = –0.72).

## Discussion

*In vitro* studies conducted on cells isolated from the anterior pituitary gland extend our understanding of neuroendocrine phenomena in mammals. The endocrine regulation of the hypothalamic–pituitary–ovarian (HPO) axis during mammalian puberty depends in large part on neuronal agents like NKB, KiSS-10 and Dyn A (1, 3, 13, 17). Neurokinin B, KiSS and Dyn A were found in identical subpopulations of neurons located in the ARC of the hypothalamus in many animal species, *e.g*. goats (29), pigs (7), sheep (6) and mice (15). It is also known that through KiSS stimulation, NKB induces GnRH release in the hypothalamus, leading to gonadotropin secretion in sheep (1, 2, 3). The role of NKB on the hypothalamus–pituitary axis in females depends on the endocrine condition. Some studies indicate that senktide, an agonist of the NKB receptor, activates LH release in adult ewes (4, 16) and rats (14). Terasawa *et al*. (26) reported that NKB and KiSS independently activated GnRH release in female rhesus monkeys before puberty, but a collaborative mechanism between these two neuropeptides accelerated the increase in GnRH secretion in pubertal females (26). In turn, the function of Dyn A involves the inhibition of GnRH release, and consequently, of LH secretion (15, 17). While the role of KiSS is better understood, the function of NKB and Dyn A at the pituitary level and their mediation of KiSS secretion at this level have not been thoroughly explored. Studies conducted on fish (female Nile tilapia) showed the effect of NKB on pituitary cell cultures, where the expression of FSHβ and LHβ mRNA was notably elevated after treatment with NKB at 10 nM and 1,000 nM (12). In addition, Mizrahi *et al*. (11) proved that an NKB analogue increased LH and FSH mRNA levels in the tilapia pituitary (11). Mijiddorj *et al*. (10) demonstrated that the NKB receptor and the Dyn A receptor were present in the LβT2 cell line (luteinising hormone subunit β T-antigen gonadotroph cells) (10). In our experiment, we demonstrated the direct *in vitro* effect of NKB and Dyn A on KiSS-10 secretion by anterior pituitary cells isolated from pubescent ewes. As shown in the present study, the effects of NKB or Dyn A on KiSS-10 secretion by ovine pituitary cells are heterogeneous and depend on the exposure time and NKB or Dyn A concentrations in the culture medium. When used at the concentrations of 10^−10^–10^−7^ M, NKB increased KiSS-10 secretion throughout the entire experiment, compared to the control. The highest level of KiSS-10 release was recorded after the 24-h exposure of the cells to NKB at the concentration of 10^−8^ M. However, no effect of NKB on the secretion of KiSS-10 was observed when it was applied at the lowest concentration (10^−11^ M). These results prove that a higher concentration of NKB contributes to the regulation of KiSS-10 secretion by pituitary cells. In addition, as indicated by our previous findings (25), the exposure of ovine pituitary cells *in vitro* to NKB at the concentration of 10^−11^–10^−7^ M resulted in elevated gonadotropin secretion. The highest level of LH secretion was observed after 24-h exposure of the cells to the concentration of 10^−8^ M, and the strongest FSH secretion came after 24-h exposure to 10^−7^ M. To date, the reports on the involvement of NKB in sexual maturation have addressed regulatory mechanisms only at the hypothalamic level. The study conducted by Topaloğlu *et al*. (27) showed that mutations in the NKB coding gene (*TAC3*) and in its receptor coding gene (*TACR3*) cause hypogonadotropic hypogonadism and block pubertal initiation (27). Our new data suggest that by increasing the secretion of kisspeptins and gonadotropins by pituitary cells, NKB may be relevant in activation of the HPO axis during puberty also at the pituitary level. Moreover, our results indicating the positive effect of NKB on the secretion of KiSS-10 and gonadotropins by pituitary cells are the basis for further studies to determine whether, as in the hypothalamus, the effect of NKB on GnRH (and subsequently LH and FSH) secretion may be mediated by kisspeptins. Whether kisspeptins mediate the secretion of these gonadotropins is better known, however, when the effect of Dyn A is considered. In our previous study we observed that Dyn A caused an increase in gonadotropin secretion at all the concentrations used (10^−11^-10^−7^ M). The LH secretion reached its maximum after 24 h in response to 10^−8^ M, and the highest FSH secretion was recorded after 24-h exposure to the lowest concentration of Dyn A (10^−11^ M) (25). Our present experiment showed no significant effect of any concentration of Dyn A on KiSS-10 secretion by ovine pituitary cells. Therefore, the effect of Dyn A on gonadotropin secretion appears not to be mediated by kisspeptins.

Unfortunately, it is difficult to compare the present results because of the lack of similar data in the literature on the mutual interactions of NKB and Dyn A at the level of the pituitary gland. A full elucidation of the role of these neuropeptides in KiSS-10 secretion at the pituitary level and the establishment of their impact on neuroendocrine regulation during puberty require further studies.

## Conclusion

The present study provides new data for the novel role of NKB in the endocrine regulation of KiSS-10 secretion from ovine anterior pituitary cells. Neurokinin B, in contrast to Dyn A, can directly affect KiSS-10 secretion from pituitary cells of pubescent ewes in a way dependent on the time of exposure to this neuropeptide and its concentration in the culture medium. Hence, our results may indicate the potential importance of NKB in the initiation of reproductive activity in ewes and, consequently, in their reaching sexual maturity in the optimal developmental window.
